# Resveratrol protects human nucleus pulposus cells from degeneration by blocking IL-6/JAK/STAT3 pathway

**DOI:** 10.1186/s40001-021-00555-1

**Published:** 2021-07-28

**Authors:** Cenhao Wu, Jun Ge, Ming Yang, Qi Yan, Yingjie Wang, Hao Yu, Huilin Yang, Jun Zou

**Affiliations:** Department of Orthopaedic Surgery, The First Affiliated Hospital of Soochow University, 188 Shizi St., Suzhou, 215006 Jiangsu China

**Keywords:** Resveratrol, Nucleus pulposus cells, IL-6/JAK/STAT3 pathway, Intervertebral disc degeneration, Proinflammatory factors, Positive feedback

## Abstract

**Background:**

Nucleus pulposus cells’ (NPCs’) degeneration is mainly responsible for the intervertebral disc degeneration (IDD), which is closely related to inflammatory response. Among the major proinflammatory factors that are related to NPCs’ degeneration, interleukin-6 (IL-6) and its downstream JAK/STAT3 pathway have received recent attention. The goal of our study is to figure out whether or how resveratrol (RSV) can protect NPCs from degeneration by affecting IL6/JAK/STAT3 pathway.

**Methods:**

Different concentrations of RSV were added to NPCs’ mediums. Cell viability was measured by MTT assay and crystal violet staining. Cell cycle and apoptosis were analyzed by flow cytometry. Protein expression level was determined by western blot. mRNA expression level was measured by qPCR.

**Results:**

Our study showed that RSV improved NPCs’ cell viability. It also inhibited cell apoptosis and cell cycle arrest, which were accompanied by the increased expression level of heat shock protein 90 (HSP90) and N-Cadherin. What’ more, RSV also improved the NPCs’ degeneration which was reflected in the increase of extracellular matrix (collagen II, Aggrecan). Moreover, RSV significantly attenuated the level of IL-6 secretion, which was accompanied by less phosphorylation of the transcription factors Janus kinase 1 (JAK1) and signal transducer and activator of transcription 3 (STAT3).

**Conclusion:**

RSV exerted its protective effect on HNPCs’ degeneration by improving cell survival and function. The possible mechanism may be associated with the suppression of JAK/STAT3 phosphorylation and the decreased IL-6 production, which could be explained by a blockage of the positive feedback control loop between IL-6 and JAK/STAT3 pathway.

## Background

It is estimated that approximately 60–80% of adults suffer from lower back pain (LBP) at least once during their lifetimes [1–3]. In addition to the pain and loss of function induced upon the patient themselves, this kind of disease imposes a great amount of economic burden on the affected family and even society. Intervertebral disc degeneration (IDD) is the pathological basis of intervertebral disc protrusion, spinal canal stenosis, unstable spine, spinal cord, and nerve root compression, and is the main cause of LBP. The current treatments for IDD can be divided into surgery-based and conservative therapies, which mainly aim to relieve symptoms. However, biological therapies focusing on a mechanism that can retard or reverse disc degeneration fundamentally are still under development. The possible mechanisms underlying IDD include various factors, such as mechanical and oxidative stress, aging, infection, obesity, blood sugar, smoking, and so on.

Many previous studies have demonstrated that inflammatory response plays an important role in the onset and progression of IDD [4]. Certain classical inflammatory cytokines, including tumor necrosis factor (TNF)-α, IL (interleukin)-1β, and IL-6, are found to be overexpressed in degenerated or herniated discs [4, 5]. What is more is that the related downstream pathways, such as AMP-activated protein kinase/Sirtuin1 (AMPK/SIRT1), phosphatidylinositol-3-kinases/protein kinase B (PI3K/AKT), and Janus kinase/signal transducer and activator of transcription 3 (JAK/STAT3), and the various therapies that target them for prevention IDD have become a hot research topic in recent times [6–9].

Resveratrol (RSV; trans-3, 5, 40-trihydroxy-trans-stilbene) is a natural phytoalexin found in peanuts, grapes, and several other plants. The anti-inflammatory, anti-cancer, anti-aging, and chondrocyte-protective effects of RSV have been demonstrated previously by many studies [10–13]. Recently, various researchers have shown that RSV can attenuate the deleterious effects on the intervertebral disc caused by external pathological factors, such as high glucose, IL-1β, and TNF-α [9, 14–16]. It has been demonstrated that RSV can effectively mitigate cell senescence and apoptosis, and can even alleviate the inflammation response caused by the aforementioned factors by regulating different signaling pathways, such as the PI3K/AKT, extracellular regulated protein kinases 1/2 (ERK1/2), and reactive oxygen species/nuclear factor kappa beta (ROS/NF-κB) pathways [8, 14, 17, 18], thus impeding IDD. Therefore, there is quite a gap—the aforementioned studies have shown a potent anti-inflammatory/anti-degenerative ability of RSV preventing IDD, though most were carried out under conditions, where nucleus pulposus cells (NPCs) were stimulated by exogenous stimulatory factors in advance, and so there are less investigations in the literature on how RSV affects NPCs at a normal physiological state. Furthermore, the mechanism of how exactly RSV affects NPCs is still a challenge for scientists globally. These are the reasons why we conducted our study and doing so, we sought to explain these matters from the perspective of the IL6/JAK/STAT3 pathway.

## Materials and methods

### Cell culture

The human nucleus pulposus cell line was purchased from ScienceCell (Carlsbad, USA, Cat. No. 4800). The detailed methods of cell resuscitation were mentioned in our previous study [19]. Transfer the tube from the liquid nitrogen to a 37 ℃ water bath. Gently shake it for about 2 min until it dissolved completely. Next, the liquid is gently blown into the centrifuge tube with 5 mL complete medium (HyClone, DMEM/HIGH GLUCOSE, cat. No. SH30243.01B). After centrifugation, the cell suspension was then transferred to the culture flask in an incubator at 37 ℃ and 5% CO_2_. The cell culture was replaced after 24 h, and the medium was changed every 2 days. When the cell adhesion rate reached 90%, the cells were passed on by 1:2.

### Experimental groups

After 2 passages, NPCs were treated with different concentrations of RSV (10 μmol/L, 20 μmol/L, and 50 μmol/L), which served as experimental groups. The control group contained no added RSV.

### Crystal violet staining

Crystal violet staining was used to analyze cell growth. The cells were digested and inoculated in 12-well plates with 2500 cells per well. The final volume of each well was 2 mL and was changed every other day. The incubation period is 7–10 days. Then discard the medium, add crystal violet solution (2 mL), and stain for 10 min. In the end, the crystal violet solution was removed. The plates were washed by PBS for 3 times, and photographed.

### MTT assay

Trypsin (0.25%) was used to digest the cell medium. The cell concentration was induced to 5–10 ×  10^5^/mL, after which cells were inoculated in a 96-well plate with 1000–10,000 cells per well at a volume of 200 μL/well. After different concentrations of RSV were added, the cells were incubated at 37 °C and 5% CO_2_ for 7 days. At different timepoints, an MTT assay (MTT Assay Kit, ab211091, Abcam, Shanghai, China) was used to measure the NPCs’ viability. The measurement procedure was as follows: 10 μL MTT solution (5 mg/mL, prepared with PBS, pH = 7.4) was added to each well. After the culture was incubated for 4 h, the supernatant was carefully removed. A total of 100 ul of DMSO to each well and the plate was oscillated for 10 min to fully melt the crystal. A 490-nm wavelength was selected to measure the light absorption value of each well, and the results were recorded.

### Analysis of the cell cycle

The detailed procedures were presented in our previous study [20]. The brief methods were as below. Trypsin (0.25%) was used to digest the cell medium. Then, centrifugate (3000 rpm) the cell culture medium for 5 min. Next, the cells were washed with precooled phosphate buffer salt (PBS). Then, centrifuge again to remove the supernatant. The cells were then fixed overnight in a solution of 70% ethanol at 4 ℃. Next, the cells are collected through another centrifuge under the same conditions, as described above. After the preparation of RNase (50 ng/mL) and propidium iodide (PI) staining solution (20 ng/mL), NPCs were digested and bathed at 37 ℃ to avoid light for 1 h. Finally, flow cytometry was used to analyze cell cycle.

### Measure of apoptotic cells

Annexin V-FITC/PI kit (HaiGene, China) was used for double staining to determine the percentage of apoptotic cells according to the instructions. The cells were washed in cold PBS and resuspended in 500 μL of binding buffer. Then, 5 μL of Annexin V-FITC solution and 5 μL of PI solution were added to each sample. The cell suspension was placed in ice, incubated in dark for 5 min, and detected by flow cytometry.

### Western blot

The growth medium was removed and the cells were washed with PBS for 3 times. The frozen RIPA lysis buffer was added into the Bio-Rad protein assay kit (Bio-Rad, Hercules, CA, USA) to extract the total protein. The same amount of protein was electrophoreted by 12% SDS-PAGE and the protein was transferred to the nitrocellulosic membrane (Bio-Rad, Hercules, CA, USA). Subsequently, the membranes were blocked at room temperature for 1 h with Tween-20 (Thermo Fisher Scientific, Waltham, USA) that contains 5% fat-free-milk and *tris*-buffered saline (TBS). After the addition of primary antibody, it was incubated at 4 ℃ overnight. The primary antibodies include anti-IL-6, anti-HSP90, anti-N-cadherin, anti-Aggrecan (Santa Cruz, Dallas, USA), anti-JAK1, anti-Phospho-JAK1, anti-JAK2, anti-Phospho-JAK2, anti-Collagen II, anti-Collagen X, anti-actin, anti-GAPDH (Abcam, Cambridge, MA, USA), anti-STAT3, anti-Phospho-STAT3 (MSD. Merck & Co., NJ, USA). On the next day, the membrane was washed and the secondary antibody (Bioworld) was added. After incubated at room temperature for 1 h, the cells were washed again with PBS. The immunoreactive protein was observed under gel imaging device (Fujifilm) with a chemiluminescence kit (Thermo Scientific). ImageJ software was used to compare the grayscale ratio of the target protein to the content of GAPDH or β-actin, and to evaluate the relative expression of the target protein.

### Reverse transcription–polymerase chain reaction

Trizol (Invitrogen, Carlsbad, USA) was used to lyse NPCs according to manufacturer's protocol. RevertAid RT Reverse Transcription Kit (TaKaRa Bio, Otsu, Japan) was used to reverse transcribed the total RNA into cDNA as instructed. SYBR Green Master Mix Reagent (Toneker, Shanghai, China) was used for quantitative PCR for determination of mRNA levels (IL-6 and STAT3) according to the protocol. An internal reference gene (GAPDH) was amplified simultaneously with the target genes. Primer sequences were as follows: IL-6-F: 5'-GAGGATACCACTCCCAACAGACC-3', IL-6-R: 5'-AAGTGCATCATCGTTGTTCATACA-3'; STAT3-F: 5'-GAGTCAGGCACTGTGGG-3', STAT3-R: 5'-CGGTCGGTTTCTGCCTGTA-3'; GAPDH-F: 5'-TTCAACGGCACAGTCAAG-3', GAPDH-R: 5'-TGGTTCACACCCATCACA-3'. The experiment was independently repeated three times.

## Results

### Resveratrol improves NPC survival

**Fig. 1:**
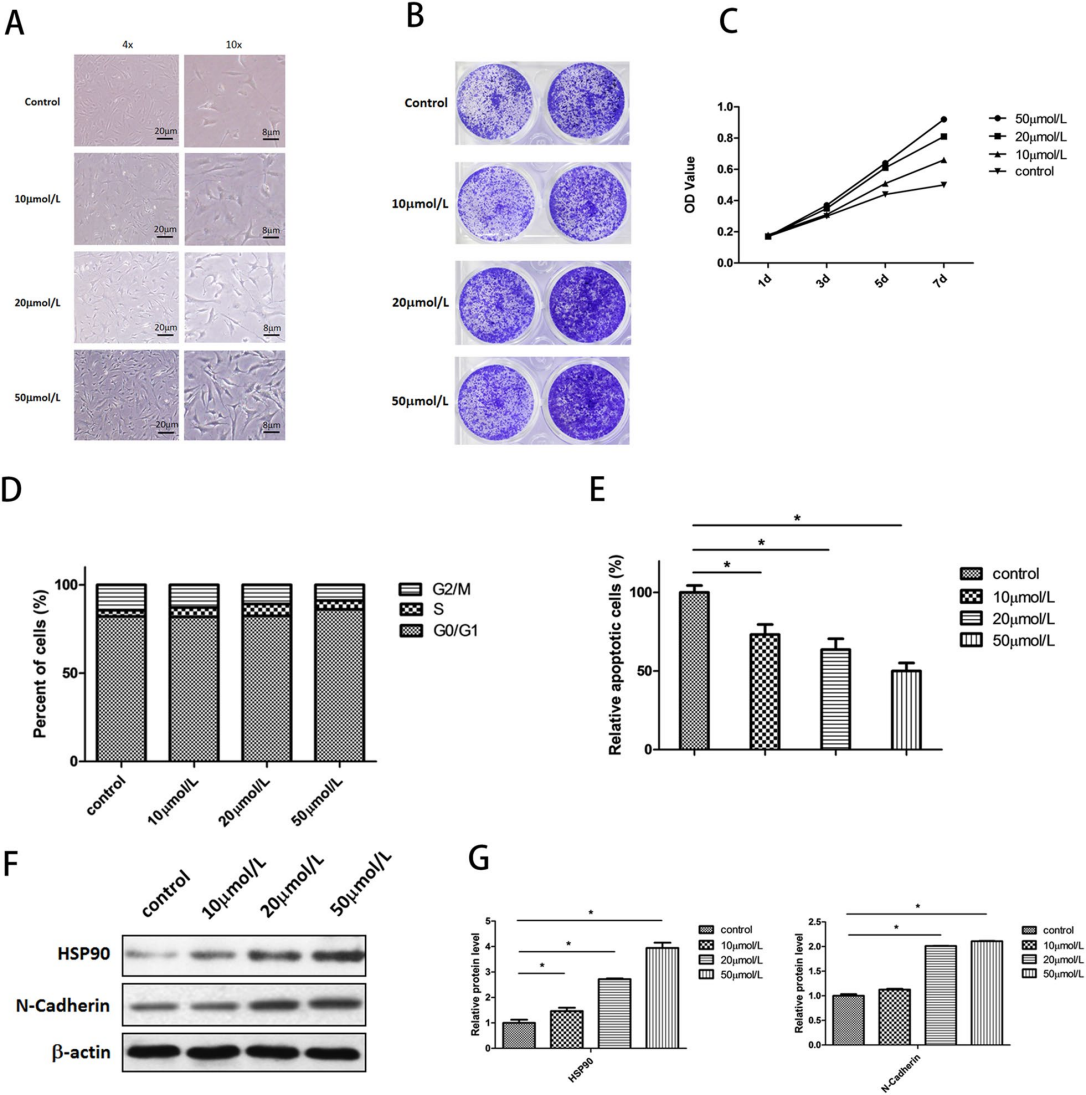
Resveratrol improved NPCs’ survival. **A** Under light microscope, cells changed from short and round to long-spindle after treated with RSV. The cell size was also enlarged in parallel with better developed pseudopodia. **B** Cell number of experimental groups increased significantly compared with that of control group. **C** MTT assay showed that RSV improved cell viability in a dose-dependent manner. **D** Cell cycle distribution showed the cell cycle transition from G2/M to G0/G1 phase after treated with RSV. **E** Flow cytometry analysis indicated that RSV reduced apoptosis in a dose-dependent manner (*p < 0.05). **F** Western blot showed that HSP90 and N-Cadherin increased when compared to the control. **G** Quantified analysis (grey value analysis) of western blot results (*p < 0.05)

After treatment with different concentrations of RSV (10, 20, 50 μmol/L), the morphology of NPCs across the four groups were observed with light microscopy. The results indicated that the cell morphology of the experimental groups changed dose-dependently when compared with that of the control group. Cells changed from round and short to long spindles after being treated with RSV. Cell size was also enlarged in parallel with better-developed pseudopodia, which meant improved cell adherence. Furthermore, cell density was potently increased after treatment with RSV (Fig. 1A). To investigate how RSV affects NPCs’ viability, we employed crystal violet staining and an MTT assay. According to the results of the crystal violet-staining assay, cell number of the experimental groups exhibited a significant increase in a dose-dependent manner compared to the control group (Fig. 1B). With the MTT assay, the results showed that the number of living cells in the experimental groups increased more rapidly in dose-dependent manner with time. When extending time, the rise in living cells in the control group tended to be mild, but in the experimental groups, it was not, also showing the potent improvement in cell viability after treatment with RSV (Fig. 1C). The analysis of the cell cycle indicated that RSV could significantly reduce the percentage of NPCs arrested in the G2/M phase and improved cell-cycle transition from G2/M to G0/G1 phase, which was reflected in the dose-dependent increase in cells at the G0/G1 phase (Fig. 1D). In addition to cell-cycle analysis, we measured the effect of RSV on NPCs’ apoptosis. The findings revealed that the decrease of NPC apoptosis was also noticeable and dose dependent after intervention with RSV (Fig. 1E). To verify the phenomenon of increased cell survival in NPCs, we tested the expression level of heat-shock protein 90 (HSP90) and N-cadherin. HSP90, which belongs to the family of stress-related proteins, is a molecular chaperone and widely involved in cell-signal transduction, hormone response, and transcriptional regulation. Moreover, it plays an important role in the survival of cells under physiological, pathological, and stress conditions. N-cadherin, which is found in nervous system, is also implicated in cell adhesion and proliferation. The result of Western blotting and the grey analysis showed that the concentrations of HSP90 and N-cadherin increased significantly in a dose-dependent manner (Fig. 1F, G). In summary, the Western blot results were consistent with the phenomenon of increased cell survival in NPCs.

### Resveratrol protects NPCs from degeneration

**Fig. 2:**
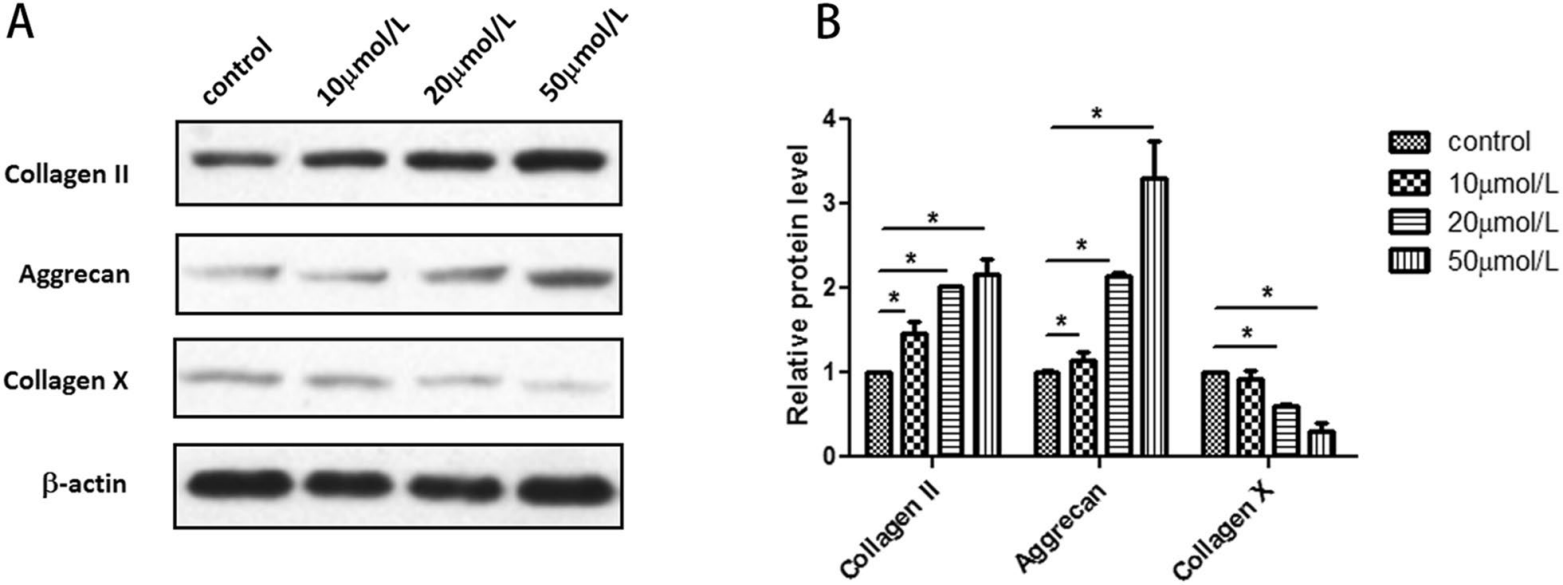
Resveratrol protects NPCs from degeneration. **A** Western blot indicated that the content of Collagen II and Aggrecan tended to increase after treated with RSV but Collagen X showed an opposite trend. **B** Quantified analysis (grey value analysis) of western blot results (*p < 0.05)

To determine whether RSV could protect NPCs from degeneration, we used a Western blot to measure the main components of NPCs’ extracellular matrix. As shown in Fig. 2A, the content of Collagen II and Aggrecan, which are closely related to the normal functioning of NPCs, was increased. Meanwhile, the content of Collagen X, which is one of the classical signs of NPC degeneration, decreased correspondingly (Fig. 2A). Grey analysis reflected how the relative protein level of Collagen II in the 10 μmol/L and 50 μmol/L groups was higher than the control group significantly. There was also a significant increase in Aggrecan expression in the 20 μmol/L and 50 μmol/L groups. As with the expression level of Collagen X, a significant decrease was shown in a dose-dependent manner (Fig. 2B).

### Resveratrol reduces the production of interleukin-6 and inhibits the activation of the JAK/STAT3 pathway, thus exerting its function

**Fig. 3:**
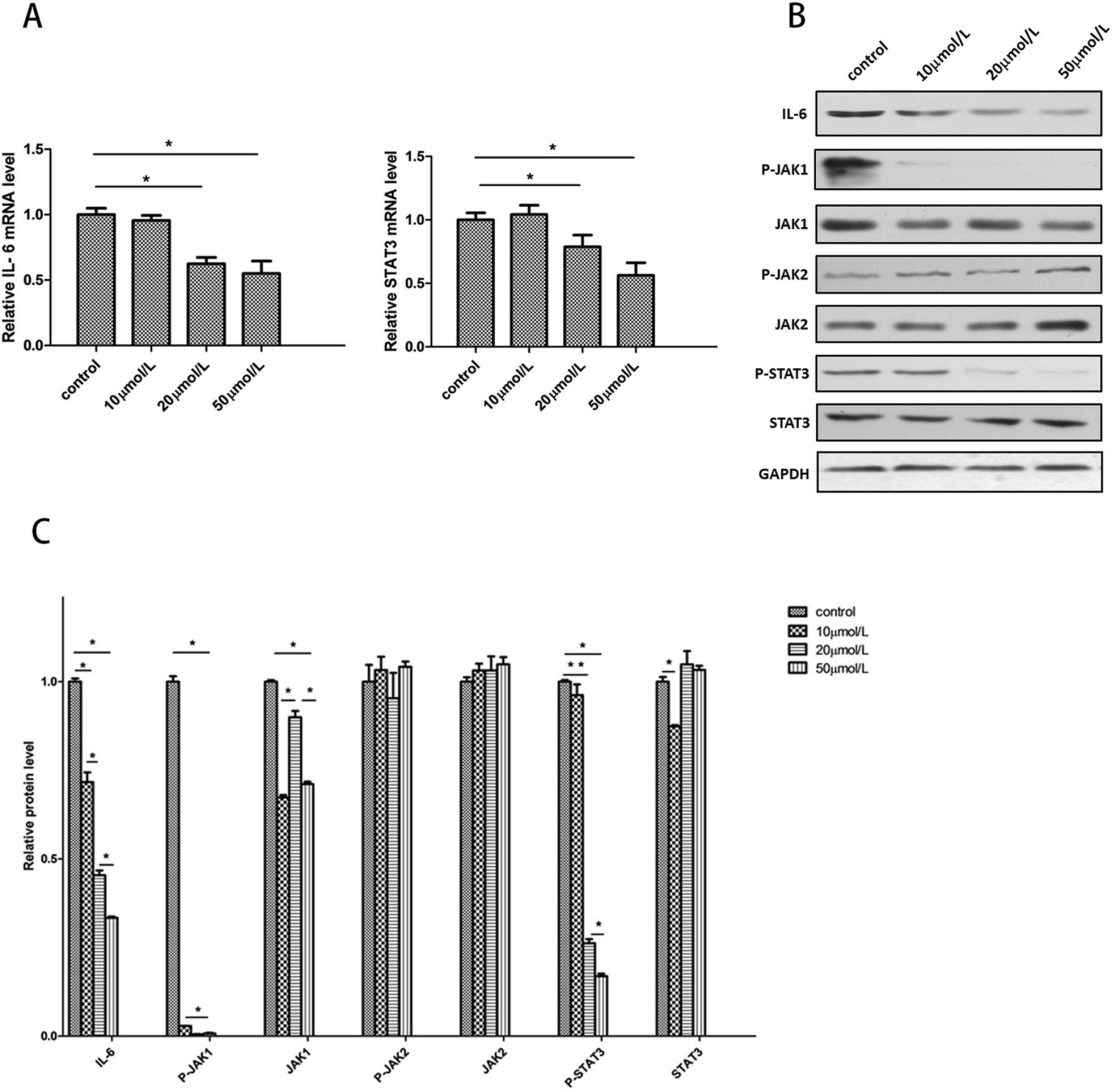
Resveratrol reduces the production of Interlukin-6 and inhibits the activation of JAK/STAT3 pathway. **A** Relative mRNA level of IL-6 and STAT3 both decreased in a dose-dependent manner (*p < 0.05). **B** Western blot of key proteins in IL-6/JAK/STAT3 pathway. The results showed that the protein level of IL-6, P-JAK1, P-STAT3 decreased more obviously than the other and decreased in a dose-dependent manner. **C** Quantified analysis (grey value analysis) of western blot results (*p < 0.05)

To explore the mechanism of RSV’s biological function, we measured the expression of two key genes in the IL-6/JAK/STAT3 pathway. The RT-PCR findings indicated that the relative mRNA level of IL-6 and STAT3 both decreased dose-dependently (Fig. 3A). For further investigation, we carried out Western blotting to measure the phosphorylation levels of the key proteins in that pathway. The results indicated that the protein levels of IL-6 decreased dose-dependently, which was consistent with the PCR results. In addition, the phosphorylation levels of JAK1 and STAT3 were both diminished after treatment with various concentrations of RSV. On the other hand, the total expression levels of JAK1, JAK2, and STAT3 seemed to be unaffected after addition of RSV. Interestingly, the phosphorylation levels of JAK2, which was another subtype of JAK, also seemed to not be affected by RSV (Fig. 3B). The results of the grey-value analysis indicated that there existed a significant dose-dependent decrease in the expression levels of IL-6, P-JAK1, and P-STAT3. A reduction of total JAK1 could also be observed, but the trend in decrease was not as obvious as with P-JAK1 (Fig. 3C). Therefore, RSV can specifically inhibit the phosphorylation of JAK1, thereby inhibiting the phosphorylation of STAT3 downstream.

## Discussion

IL-6, recognized as one of major pro-inflammatory cytokines, such as IL-1 β and TNF-α, belongs to the family of IL-6-type cytokines which includes IL-6, leukemia inhibitory factor 9 (LIF), IL-11, ciliary neurotrophic factor (CNTF), oncostatin M (OSM), cardiotrophin-like cytokine (CLC) and cardiotrophin-1 (CT-1). These aforementioned cytokines are mainly involved in activating target genes responsible for cell differentiation, survival, proliferation, and apoptosis [21]. After IL-6 is bound to IL-6R (IL-6 receptor α on the cell membrane, CD126) or sIL-6 (the soluble form of IL-6R), the ligand–receptor complex will associate with glycoprotein 130 (gp130), promoting dimerization and the subsequent initiation of intracellular signaling. The three major downstream signaling pathways include the MEK/ERK, PI3-K/AKT, and JAK/STAT3 pathways [22]. When considering the role of inflammatory cytokines in inducing NPC degeneration, IL-6 receives less attention than IL-1β and TNF-α, though they all belongs to the family of proinflammatory cytokines. It is known that some classical inflammatory cytokines, such as TNF-α, IL-1β, and IL-6, are found to be overexpressed in degenerated or herniated discs, but IL-6 is not so ‘classical’ than the other two, because the expression levels of IL-6 are demonstrated to be affected by IL-1β or TNF-α, especially when the other two are applied to induce NPC degeneration. In addition, researchers have become more willing to view this as a ‘sign’ of inflammatory response or it being a ‘participant’, just like metalloproteases (MMPs) but not an ‘inducer’, such as IL-1β or TNF-α. Rebecca et al. found that after addition of exogenous IL-6 and IL-6 soluble receptor, the effects of which had been induced by IL-1β or TNF-α on NPCs were significantly enlarged [23]. Therefore, what exactly the role of a dose of IL-6 play in IDD remains a question. Actually, IL-6 itself and its downstream JAK/STAT3 pathway has already been shown to be involved in the pathogenesis of IDD in recent years [24]. In that study, the activation of the JAK/STAT3 pathway can directly increase the expression levels of cyclooxygenase-2 and matrix metalloprotease-13, thus causing IDD without the involvement of ‘classical’ IL-1β or TNF-α. Thus, with further unraveling of the mechanism of IL-6 and its downstream pathways in recent years, we potentially must look more closely at them for their involvement in the initiation or progression of IDD in the past. Therefore, in terms of RSV, there is no need to doubt its therapeutic impact on IDD, because there have been plenty of studies demonstrating that in the past. However, nearly, all of them have described the mechanism from the view of inhibiting the downstream pathways that have already been activated by IL-1β or TNF-α [8, 9, 16]. We definitely do not deny that, but we want to explain the therapeutic mechanism from a novel angle, which is IL-6 and the JAK/STAT3 pathway, the reason why we have concentrated on them and established meaningful findings.

IL-6 itself can be induced by various stimuli that mostly exert their functions through the activation of NF-κB, CREB, C/EBP, and AP-1, which are transcription factors shown to bind to IL-6 promoter [25–27]. As such, IL-6 exhibits a special characteristic, whereby it can be auto-regulated [28, 29]. Studies have found that certain downstream pathways triggered by IL-6 can sometimes act upstream, thus regulating the expression of IL-6. For example, MEK/ERK kinase does this by activating NF-κB and PI3-K/AKT pursues this purpose by activating IKK-α, which in turn activates AP-1 and NF-κB [30–32]. However, the JAK/STAT3 pathway, as the most well-known IL-6 downstream pathway, remains controversial until Huang et al. claimed that JAK/STAT3, in combination with other downstream IL-6 pathways, frequently and significantly promoted IL-6 autocrine through a positive feedback loop in a wide range of cancer cell lines and clinical samples [33]. The phenomenon of feedback regulation of IL-6 in cancer cells has received more attention than that in NPCs for many reasons. There is no such official statement that type of phenomenon exists in NPCs, not to mention its role in IDD. However, it is possible to still find certain clues to indicate the existence of the phenomenon in NPCs. For example, the expression levels of IL-6 and the JAK/STAT3 pathway are elevated in degenerative lumber disc tissues, which have often been observed in various cancer tissues [6]. Interestingly, in the study of Rebecca et al., the IL-6 expression levels of NPCs were also elevated after exogenous IL-6 was added [23]. Yet, it did not occur to them that this could be a clue to demonstrate the existence of positive feedback regulation of IL-6 in NPCs at that time.

Our study has shown that the phosphorylation levels of JAK1/STAT3 and the expression levels of IL-6 are both decreased after treatment with different concentrations of RSV, and that could be explained by the blockage of a positive feedback loop of IL-6/JAK/STAT3 by RSV. Though this supposedly makes sense to some degree, we still do not know if RSV may also inactivate the upstream pathways of IL-6, even when IL-1β or TNF-α is not added to NPCs. To our knowledge, we have been the first to investigate the biological function of RSV regarding NPCs under relatively normal physiological conditions. In the future, of course, clinical trials are mandatory to examine the therapeutic effect of RSV in IDD treatment. What’s more, considering RSV’s excellent anti-inflammatory effects, it is likely to be applied to some patients with other kinds of degenerative or inflammatory axial and articular pathologies. We hope our findings could be meaningful in terms of further investigation surrounding the mechanistic study of RSV, especially as that pertains to NPCs.

## Conclusion

In conclusion, resveratrol improves the biological behavior of NPCs in multiple ways. First, we found that resveratrol improves cell survival by attenuating cell-cycle arrest and reducing apoptosis. Second, resveratrol promotes the synthesis of the extracellular matrix. The mechanism of these effects may be associated with the suppression of JAK/STAT3 phosphorylation and decreased IL-6 production, which could be explained by a blockage of the positive feedback control loop between IL-6 and the JAK/STAT3 pathway.

## Data Availability

All the data and materials are available.
